# Drug Prescription in the Department of Medicine of a Tertiary Care Hospital according to the World Health Organization/International Network for Rational Use of Drugs Core Indicators: A Descriptive Crosssectional Survey

**DOI:** 10.31729/jnma.5612

**Published:** 2021-08-31

**Authors:** Jyoti Tara Manandhar Shrestha, Saurabh Tiwari, Dilip Kumar Kushwaha, Pratigya Bhattarai, Ruchi Shrestha

**Affiliations:** 1 Department of Pharmacology, Kathmandu University School of Medical Sciences, Dhulikhel, Kavrepalanchok, Nepal; 2 Kathmandu University School of Medical Sciences, Dhulikhel, Kavrepalanchok, Nepal

**Keywords:** *drug utilization*, *drug combinations*, *essential drugs*, *prescriptions*

## Abstract

**Introduction:**

To establish appropriate health care services in developing countries, rationalization of drug use holds utmost importance. Drug use patterns can be found out using Core Indicators of the World Health Organisation in collaboration with the International Network of Rational Use of Drugs. With the help of the indicators, this study aimed to find out the way the drugs were prescribed in the Medicine out-patient department of a tertiary care hospital.

**Methods:**

A descriptive cross-sectional survey was conducted from October 2019 to March 2020 in a tertiary care hospital. The ethical approval was taken from the Institutional Review Committee of the Dhulikhel hospital (reference number 198/19). Convenient sampling was done. After taking consent from the patient, data was collected from prescriptions written on the patient's card. The data were analysed using Statistical Package for the Social Sciences Version 25. Descriptive statistics were applied and the results were expressed as frequency and percentage, mean and standard deviation.

**Results:**

A total of 559 prescriptions were analysed, of which a total of 1427 medicines were found to be prescribed with an average number of medicines per the prescription of 2.55±1.388. Drugs prescribed by generic name were 820 (57.5%), antibiotics were 138 (9.7%) and injections were 8 (0.6%). Drugs prescribed from the Essential Drug List of Nepal was 939 (65.8%).

**Conclusions:**

Our study revealed that despite some results being up to the mark, there is a requisite for the proper regulation of prescribing and dispensing drugs in order to promote rationalisation.

## INTRODUCTION

Rational prescribing is of fundamental importance to achieve a proper quality of health care for patients. It is estimated that more than 50% of medicines are inaccurately prescribed, sold, dispensed and patients fail to take them properly.

The irrational practice of drug use is a grave concern as it leads to ineffective and cost-prohibitive treatment, wastage of resources, increased adverse effects and drug-resistance.^1^ Irrational prescribing includes polypharmacy, unnecessary use of antimicrobials and injections including the use of brand names.^2^ In developing as well as developed countries, there are numerous cases of drug misuse every so often. To comprehend the trend of prescribing patterns, the World Health Organization (WHO) in collaboration with the International Network of Rational Use of Drugs (INRUD) developed standard indicators.^3^

In this study, using these prescribing indicators we aimed to find out the use of drugs according to WHO/INRUD core indicators in Dhulikhel Hospital.

## METHODS

A descriptive cross-sectional survey was conducted in the medicine outpatient department (OPD) of Dhulikhel Hospital, Kavrepalanchok from October 2019 to March 2020 after getting ethical approval from the Institutional Review Committee of Kathmandu University School of Medical Sciences (KUSMS) (Reference number: 198/19). The patients attending medicine OPD during the period of 10:00 AM to 12:00 Noon and 4:00 PM to 6:00 PM on an alternate day basis except on public holidays were selected for the study. Patients planned for admission, prescribed with vaccines and patients not willing to participate were excluded. Convenient sampling was done and the sample size was calculated as,

n = Z^2^ × p × q / e^2^

  = (1.96)^2^ × (0.5) × (1-0.5) / (0.05)^2^

  = 386

Where,

n = minimum required sample size
Z = 1.96 at 95% Confidence Interval (CI)
p = prevalence taken as 50% for maximum sample size
q = 1-p
e = margin of error, 5%

Adding a 10% non-response rate, the sample size of 422 was reached. But we collected data from 559 patients. The data were collected using the Prescribing Indicator developed by WHO/INRUD. The prescription form of 559 patients were chosen and descriptive parameters like mean, standard deviation, frequency and percentages were calculated using Statistical Package for the Social Sciences version 25 and presented in tables and figures.

## RESULTS

Total 559 prescriptions were collected and analyzed, 238 (42.6%) male and 321 (57.4%) females were included in our study, which had the mean age of 44.3±16.23 years ranging from 17 to 85 years visited medicine OPD during our study period.

**Figure 1:**
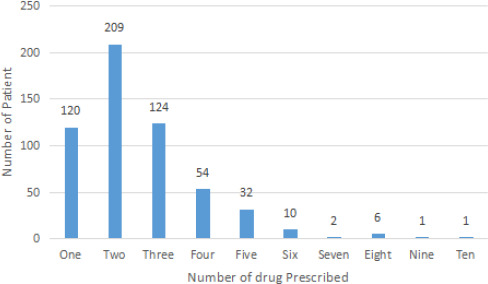
Number of drugs prescribed per patient assuming each prescription as an individual.

A total of 1427 medicines were found to be prescribed. Thus, the average number of medicines per prescription was calculated to be 2.55 (Figure 1). Drugs prescribed by generic name were 820 (57.5%) and antibiotics prescribed 138 (9.7%). Moreover, 939 (65.8%) of prescribed drugs were from the Essential Drug List (EDL) of Nepal. Additionally, 190 (13.3%) of prescriptions had at least one Fixed-Dose Combination (FDC). Only 8 (0.6%) patients, assuming each prescription as an individual, were prescribed injection.

Drugs for cardiovascular disorders, peptic acid disorder and analgesics were the most commonly prescribed which was evaluated to be 300 (21.0%), 167 (11.7%) and 155 (10.9%) respectively (Table 1).

**Table 1:** Drugs prescribed for different ailments.

Drugs	n (%)
Drugs for cardiovascular disorders	300 (21.0)
Drugs for acid-peptic disorders	167 (11.7)
Analgesics	155 (10.9)
Antimicrobials	138 (9.7)
Antidiabetics	124 (8.7)
Hormones	108 (7.6)
Bronchodilators	79 (5.5)
psychiatric & neurological disorders	57 (4)
Antihistaminics	43 (3.0)
Cough and cold remedies	30 (2.1)
Antispasmodics	32 (2.2)
Prokinetic and antiemetics	30 (2.1)
Multivitamin preparations	74 (5.2)
Others	90 (6.3)

## DISCUSSION

This study using WHO/INRUD prescribing indicators and division of the drugs into different categories help to shed light on the prescribing behaviour of a tertiary care hospital in Nepal.

In our study, we found that female patients (58.7%) were greater than male patients (41.3%). The trend was observed to be as such to other similar studies conducted by Chapagain, et al., Karki, et al., Saraff, et al. and Praveen et, al.^4-6^

The average number of drugs per encounter in this study was found to be 2.55, which is similar to the results of studies conducted in UAE ^7^and Nepal ^5^ being 2.49 and 2.60 respectively. But it was found to be less than that reported in the other two tertiary care hospitals in Nepal ^6,8^ being 4.68 and 5.85 respectively. Overprescribing than necessary leads to polypharmacy. There are several negative consequences of polypharmacy like increased cost of health care, drug interactions, non-adherence to the drug therapy and increased risk of adverse drug events.^9^ Hence, it is always preferable to keep the mean number of drugs per prescription as low as possible.^10^

Prescribing drugs using generic names is an ideal practice because it reduces medical confusion, promotes clarity amongst healthcare providers, aids hospital pharmacies to have proper inventory control and provides a selection of more drug alternatives. It also significantly lowers the inappreciable profit margin put by pharmaceutical companies owing to drug promotion considerably minimising the overall cost of treatment. Thus, it is arguably "the best practice" as believed by many national and international organisations for rationalising drugs.^2,3^ Our study shows that a total of 820 (57.5%) drugs are prescribed by generic name which demonstrates considerable value with respect to other data.^6,8,11^ However, prescription by generic name varies widely across Nepal, only in province no. 1, Saraff, et al. reported that there was zero prescription by generic name whereas Kumud, et al. reported it to be 45.18%.^4,6^ Furthermore, WHO states that an average of 44% of drugs in health units of Nepal were prescribed using generic names.^2^ The analysis above suggests that there is no proper implementation of generic name prescribing policy in Nepal as it differs widely.

WHO considers antibiotic resistance to be one of the biggest threats to global health, food security and development as of today. It occurs due to misuse, overuse and over-prescription of antibiotics.^12^ But, the main cause of antibiotic resistance is antibiotics themselves. Therefore, it is vital to address this issue by rationalising the use of antimicrobial drugs. WHO recommends the optimal value of antibiotics used should be within 15-25%.^1^ This study demonstrated that the percentage of antimicrobial drugs was to be 138 (9.7%) which was remarkably lower than the suggested value. This may be due to the location of the conducted research being an out-patient medicine ward. In contrast, in similar studies conducted by Shrestha et al., Chapagain et al., Begum et al. and Dahal et al., the data reported were tremendously higher than the standards reporting 64.1%, 40.44%, 70.33% and 57% respectively.^4,8,11,13^ However, our pattern was alike reported values by Mahamood, et al. and Karki, et al. which were found to be 9.8% and 11.7% respectively.^5,7^

The prescriptions consisting of injections were only 8 (0.6%). The outcome was extremely low compared to other studies.^4,6,11^ Use of injections can increase the overall risk of spreading communicable diseases, especially blood-borne diseases. Due to this reason, it is better to keep the value of injection prescriptions as low as possible.

According to the definition given by WHO, essential medicines are those that satisfy the priority health care needs of the population. Everyone should have access to them in adequate amounts and doses with assured quality and affordable cost.^2^ In our study, we found that 65.8% of prescribed medicines were from the Essential Medicine List of Nepal, 2016 which contains 359 medicines. Our result was found to be lower than that of the study conducted in Primary Health Care Facilities by Dahal, et al. which was found to be 85.19%.^13^ This is because EML is developed specifically for PHC. However, it is encouraged to prescribe drugs from EDL also in Tertiary Health Care Facilities because of the overall benefit. Moreover, studies conducted in other Tertiary Health Care Facilities, Shrestha, et al. and Karki, et al. reported 47.60% and 34.3% respectively.^5,8^ Comparing these results with our result, it was found that we had more drugs prescribed from EDL.

Our study reports 14.12% of drugs prescribed were Fixed-Dose Combinations. A tertiary care hospital in western Nepal reported a slightly higher value of 21.67%.^14^ The use of FDC increases patient compliance, greater efficacy, reduces cost and side effects. Despite this, FDC may lead to an inflexible ratio as the marketing strategy of pharmaceutical companies leading to increased toxicity. Thus, these pros and cons should be taken into account while prescribing drugs.

The most commonly prescribed drug in this study was for cardiovascular disorders 300 (21.0%) followed by drugs used for acid peptic disorders 167 (11.7%), analgesics 155 (10.9%), antibiotics 138 (9.7%), hormones 108 (7.6%) and multivitamins 74 (5.2%). Whereas, a study conducted in a tertiary hospital of province no.1 by Saraff, et al. in the medicine ward had results with the most common drug being gastrointestinal tract drugs (29.45%) followed by antimicrobial agents (21.21%) and cardiovascular drugs (17.98%). However, the prescribed percentage of multivitamins reported by Saraff, et al. (5.64%) was similar to our study.^6^

This study was mono-centric which may not demonstrate the situation of the whole nation. This result may be unable to reflect the trend of prescription of all seasons as it was conducted from October 2019 to March 2020. Having a limited sample size was a major drawback of this study. The main challenge was to carry on the study because a lockdown was imposed since March. It also drastically reduced the patient flow in the medicine out-patient department.

## CONCLUSIONS

The study was conducted to reflect the prescribing pattern of Medicine OPD of Dhulikhel Hospital using WHO/INRUD indicators. Our study calculated the number of drugs used per prescription lower than those calculated by other similar studies. Generic names and Essential Drug List used in the prescription were revealed to be comparatively higher in practice than those revealed by other similar studies conducted in different hospitals in Nepal but they are still below the optimum percentage as per WHO. Thus, prescribing with a generic name and prescription of EDL must be encouraged with the implementation of some robust policy at a hospital, national and international levels to promote the rational use of drugs. Moreover, we should acknowledge antibiotic prescribed percentage and injection prescribed percentage which are considerably lower than those recommended by WHO which prevent drug resistance. Therefore, promoting the utilisation of healthcare resources.
